# A UK national cross-sectional survey of stroke support groups: exploring the role of social identification and group processes in reducing loneliness

**DOI:** 10.1186/s12889-024-20432-w

**Published:** 2024-10-29

**Authors:** Laura Hollands, Raff Calitri, Catherine Haslam, Ruth A. Lamont, Luke Mounce, Mark Tarrant

**Affiliations:** 1 University of Exeter Medical School, St Luke’s Campus, Exeter, EX1 2LU UK; 2 School of Psychology, University of Queensland, St. Lucia, QLD 4072 Australia; 3 Natural England, Foss House, York, YO1 7PX UK; 4 School of Psychology, University of Plymouth, Plymouth, PL4 8AA UK

**Keywords:** Social identity, Stroke, Peer support, Loneliness, Group processes, Group dynamics, Social support, Support group, Group-based interventions

## Abstract

**Background:**

Loneliness is a common experience following stroke. Stroke support groups may protect against loneliness, but little is known about how these groups exert their influence. This research drew upon current theorising on the role of groups for health and explored i) social identification as a potential mechanism for overcoming loneliness, and ii) psychological group resources (support, control, self-esteem), and functional group processes (clear goals, group autonomy, member continuity) which might structure social identification.

**Methods:**

Five hundred seventy-nine stroke survivors from 84 Stroke Association support groups across the UK completed a cross-sectional survey measuring: support group identification; psychological resources (given and received social support, control, self-esteem, identity centrality); functional processes (goal clarity, group autonomy, member continuity); and loneliness (3-item UCLA Loneliness Scale).

**Results:**

Greater support group identification was associated with reduced loneliness (β = -0.45, *p* < 0.001). Given (β = 0.17, *p* = 0.001) and received (β = 0.10, *p* < 0.001) social support, goal clarity (β = 0.17, *p* = 0.002), and group member continuity (β = 0.19, *p* < 0.001) were all associated with greater support group identification.

**Conclusions:**

Social identification with the group may be a mechanism by which stroke support groups alleviate loneliness, potentially through facilitating attendance, mutual social support and the development of collective goals. Further research should explore how these processes influence social identification in newly formed groups, where social identity has not yet been established.

**Supplementary Information:**

The online version contains supplementary material available at 10.1186/s12889-024-20432-w.

## Introduction

Managing loneliness in many health conditions and contexts is key to supporting rehabilitation [1]. In 2019, global prevalence of stroke was 101 million, amounting to 143 million disability-adjusted life years lost due to its concomitant disabilities [2]. Physical mobility, speech and comprehension impairments are common and present major challenges to stroke survivors’ capacity for social participation [3], resulting in pervasive disconnection and loneliness among stroke survivors [4]. Indeed, stroke survivors describe a sense of”identity aloneness” in social settings, fuelled by the sense that they are different from those around them [5]. Post-stroke loneliness is associated with psychological distress [6], poorer life satisfaction [1], depression [7, 8], anxiety [9], poorer functional and societal participation outcomes [10, 11], and mortality [12]. Together these impacts can undermine recovery from stroke.

In response to these difficulties, stroke support groups, such as those offered by the UK Stroke Association (SA), are offered to increase opportunities for survivors to connect and access to informational, practical, and emotional support as they adjust to life after stroke. Participation in such groups has been associated with reductions in social isolation [13], access to social support [13, 14], improved confidence, enhanced health and wellbeing [13] and greater a sense of “being understood” [14]. Recognising this potential, the National Institute for Health and Care Excellence (NICE, [15]) recommends that health services facilitate stroke survivor participation in dedicated community groups as part of their wider rehabilitation.

However, the effects of participation in such interventions are mixed. For example, although social support is often stated as a core motivator for providing group-based care, social support interventions for stroke [16] and other health conditions [17] do not always yield positive health outcomes [18]. And, more generally, in research where participation in a group intervention is associated with a range of positive health outcomes, there remains marked heterogeneity across individual studies in the extent to which this is observed [19, 20].

To advance our understanding of when and why individuals experience these psychosocial benefits, we need to identify the mechanisms through which support group membership functions as a resource for reducing loneliness in stroke survivors. Research suggests that it is not simply being part of a social group, but *how* group members cognitively and emotionally experience a group that is instrumental in deriving health benefits from it. The Social Identity Approach to Heath [21, 22] recognises the fundamental role that group membership — as "us” stroke survivors — plays in an individual’s subjective sense of self or identity [23]. When individuals define themselves as part of a group, they internalise the norms, values, and resources of that group which, in turn, become important drivers of feelings and behaviour [24]. For members of stroke groups, identifying as a stroke group member may encourage them to adopt the group’s positive norms (around overcoming stroke-related challenges), values (such as looking out for others), and accepting emotional and practical support. At a time when stroke survivors may have lost other important social identities (e.g., relating to work, physical activity, cognitive and social skills), identification with a stroke support group might have the potential to support self-esteem and rehabilitation. However, the Social Identity Approach to Heath predicts that it is only to the extent that an individual *subjectively* identifies themselves as a group member that they will engage with the group (e.g., to enact the group’s norms and values) and benefit from its resources [24]. This speaks to the first question in our research: does social identification with a stroke support group lead to reduced loneliness among stroke survivors?

Our second question concerns *how* such identification reduces loneliness. First, as noted above, social identification enables access to a range of health-promoting resources such as access to social support [25], and an enhanced sense of personal control [26] and self-esteem [27]. Evidence showing the importance of these resources for psychosocial health outcomes has been observed across a range of long-term health conditions (e.g., in eating disorders, [28], depression, [29], and obesity, e.g., [30]). For stroke survivors, such resources may be critical for supporting adjustment to disability and maintaining wellbeing. However, physical and cognitive disability associated with the condition can impede development and maintenance of social relationships [1, 5]. Stroke support groups may provide an essential basis for provision of these resources that stroke survivors may struggle to access elsewhere. Furthermore, the shared understanding of stroke that these groups are founded upon may provide a context for deeper, meaningful connection around which members can build a shared social identity and, through this, come to represent a rich source of resources that support recovery, to build quality social relationships that reduce feelings of loneliness. Access to these resources might also reinforce identification with the group, in that they reaffirm that the group is a source of valuable and meaningful relationships, thus keeping members engaged with long-established groups beyond the start of recovery.

Beyond access to group resources, however, it is clear that other factors also affect how groups function. Group members who work together towards common goals [30–32], can influence the group [33–35], and routinely commit to the group and its activities [36], have a strong sense of the nature, function, and value of that group. Evidence to support this point comes from research in clinical contexts — in studies examining support groups for obesity management [36], aphasia choirs [32], and care homes [33] — all enabled through group identification. These factors contribute to shaping and further strengthening identification by laying the groundwork for group function, member interaction, and members’ perceived role within the group. Thus, by shaping identification, these functional processes may impact upon a member’s connection with the group and ultimately upon loneliness. In this research we test the combined contribution of group resources and functional processes to examine what may shape social identification in existing support groups where membership is long established. All this speaks to our second research question: are group resources and functions a basis for strengthening stroke support group identification?

### Study aims

The aim of this study was to: 1) examine the contribution of strength of group identification to loneliness outcomes in members of stroke support groups and, 2) explore the role of group resources (given and received social support, control, self-esteem, identity centrality) and functions (goal clarity, group autonomy, group member continuity) in strengthening stroke support group identification.

## Materials and methods

### Study design

This cross-sectional study comprised a national survey of the UK Stroke Association’s (SA) network of peer-led support groups. Data were collected between November 2019 and March 2020. The survey was initially designed for a longitudinal study of stroke support group members. However, due to the COVID-19 pandemic the follow-up survey was adjusted to reflect the context of stroke group membership at that time. Findings from that survey are reported elsewhere [37]. Our stroke advisory group, which comprised stroke survivors, stroke group leaders, and an aphasiologist, advised on acceptability of survey content, study materials (information sheets, consent forms), and recruitment processes.

### Study population

The SA peer support groups are led by volunteer members who may themselves be stroke survivors. A convenience sampling approach was taken to data collection, where all groups in the SA network were invited to take part (*N* = 214). Each group leader was contacted and asked to share information about the study with their groups. At least one week later, the group leaders distributed the consent form and a questionnaire during one of the group’s regular meetings, which participants completed themselves. The group leader returned the questionnaires to the study team by post. Aphasia-friendly versions of the questionnaire and study documents were available for participants that required these. As the SA does not maintain official membership numbers for each group, we requested group leaders to estimate the number of questionnaires needed. Consequently, the exact number of stroke survivors approached could not be accurately recorded. Group members were eligible to take part if they were (i) a stroke survivor, (ii) a member of a Stroke Association peer support group, and (iii) over 18 years of age.

### Measures

Variable selection was informed by prior research evidence and was underpinned by the social identity approach to health. None of the measures have been formally validated for use with stroke survivors but where possible, measures validated in other populations were used. For example, despite not being validated for stroke, the 3-item UCLA loneliness scale has been successfully utilised in other surveys conducted with stroke survivors [38]. In cases where validated, brief measures for a group process were not available, bespoke single-item measures were created by the study team.

#### Demographics

The following demographic variables were collected: age, gender, living alone, time since stroke, perceived health (poor, fair, good, excellent; as a proxy for level of disability [39]), peer support group role (volunteer or member), length of group membership, group meeting frequency, and frequency of group attendance.

#### Loneliness

Loneliness was measured using the three-item version of the UCLA Loneliness Scale (α = 0.88) [40]: “How often do you feel that you lack companionship?”, “How often do you feel left out?”, “How often do you feel isolated from others?”. Responses were scored on a 3-point scale “Hardly ever”, “Some of the time”, “Often”. Scores were summed (range 3 – 9) with higher scores indicating greater levels of loneliness.

#### Group identification, psychological resources and functional processes

A four-item measure of group identification (α = 0.81) [41] was used: “I feel a bond with my group”, “I feel similar to other group members”, “I have a sense of belonging to my group”, and “I have a lot in common with the members of my group”. All items were scored on a five-point Likert scale, from “completely disagree” to “completely agree”. An average score was taken if two or more items were completed (otherwise data were considered missing; range 0 – 4), with higher scores indicating stronger group identification.

##### Group resources

Received social support was based on the 3-item Oslo Support Scale measure (α = 0.72) [42]: “How many people in the group are so close to you that you can count on them if you have great personal problems?”, (none, 1–2, 3–5, 6 +) “How much interest and concern do people in the group show in what you do?” (none, little, uncertain, some, a lot), and “How easy is it to get practical help from the people in the group if you need it?” (very difficult, difficult, possible, easy, very easy). Scores were summed (0 – 11); higher scores indicate greater access to social support.

Given social support was measured by the single item: “I give emotional or practical support to other group members” and was scored on a five-point Likert scale, from “completely disagree” to “completely agree”.

Control was measured using the 3-item measure (α = 0.83) [43] “I feel in control of my life”, “I am free to live my life how I wish”, and “My experiences in life are due to my own actions”, each scored on a five-point Likert scale, from “completely disagree” to “completely agree”. An average score was taken (0 – 4), with higher scores indicating stronger sense of control.

Self-esteem was measured using a single-item measure [44] “I have high self-esteem” and was scored on a five-point Likert scale, from “completely disagree” to “completely agree” (range from 0 – 4).

Identity centrality was captured using the single item “I often think about the fact that I am a stroke survivor” [45] scored on a five-point Likert scale, from “completely disagree” to “completely agree”.

##### Group functions

Having clear group goals was measured using a single item taken from the multi-item measure [46] “We have clear group goals” to reflect a sense of clear collective group purpose, and was scored on a five-point Likert scale, from “completely disagree” to “completely agree”.

Group autonomy was measured using a single item adapted from Langfred [47] multi-item measure “We are able to influence the sessions and activities of the group”, and was scored on a five-point Likert scale, from “completely disagree” to “completely agree”.

Group member continuity was measured using the single item “Do the same people come to each session?”, scored on a five-point Likert scale (never, rarely, sometimes, often, most of the time).

For all multi-item measures, Cronbach’s alpha was calculated from the present data.

### Data analysis

For the single item measures, where a response option (e.g., “completely disagree”) contained less than 10% of responses for that item, response categories were merged. As such, the single item process measures of given social support, stroke survivor identity centrality, goal clarity, group autonomy and member continuity, were dichotomised to “Strongly disagree/Disagree/Neither agree nor disagree” and “Agree/Strongly agree”.

Descriptive data are summarised using means and standard deviations for normally distributed data, and proportions (N, %) were used for dichotomous variables. Hypotheses were tested using multilevel regression modelling, to account for the clustered nature of the data — individual responses are level 1, nested within support groups at level 2. To address RQ1, support group identification and demographic covariates were first run individually in univariable models predicting loneliness to provide unadjusted estimates. To obtain adjusted estimates, support group identification was entered into a multivariable model predicting loneliness, alongside all demographic covariates for which there was at least weak evidence of an association (*p* < 0.10). Similarly, to address RQ2, each predictor (group resources: given and received social support, control, self-esteem, identity centrality, and group function: goal clarity, group autonomy, and group member continuity) and demographic covariates were first run individually in univariable models predicting support group identification to provide unadjusted estimates. To provide adjusted estimates of the contribution of these predictors to identification, all predictors and demographic covariates for which there was at least weak evidence of an association (p < 0.10) were entered into a multivariable model. Data are presented as unstandardized beta coefficients.

Initial covariates for both research questions were theoretically derived. Alpha was set at 0.05. The multiple relationships tested were prespecified based on theory, hence data were not corrected for multiple comparisons and results should be interpreted in light of this. Individual item data missingness sat at or below 5%, and therefore a complete case analysis was pursued. Exploratory multiple imputation of missing variables was conducted, and the same models run; results did not differ in terms of the strength of evidence for effects (estimates are provided in Appendix A).

### Ethical approval

This study was performed in accordance with the Declaration of Helsinki. Ethical approval was granted by the University of Exeter College of Medicine and Health Research Ethics Committee (Oct19/B/223). Informed consent to participate in this study was obtained from all participants.

## Results

### Sample characteristics

The survey was completed by 579 stroke survivors; 446 people completed the standard questionnaire and 133 completed the aphasia-friendly questionnaire. Responses represented 84 peer support groups across England, Scotland, Wales and Northern Ireland, with between one and 25 questionnaires completed per group (median = 6).

**Fig. 1:**
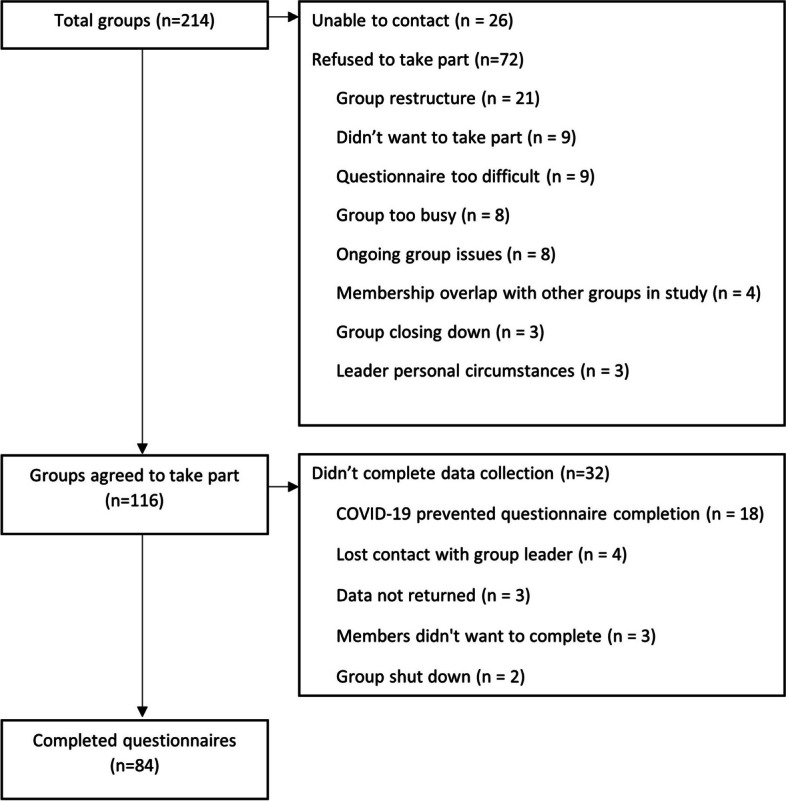
Stroke support group recruitment and reasons for non-participation

Group recruitment and reasons for non-participation are presented in Fig. 1. Of the 214 groups potentially eligible for inclusion, 26 could not be contacted and 72 refused to take part. The main reason for refusal was due to the organisational restructuring of support groups taking place across a number of Stroke Association local hubs. One-hundred and sixteen groups agreed to take part. Of these, 32 groups did not complete data collection. Half of these groups could not complete data collection due to the start of the COVID-19 pandemic preventing the group from meeting. As the SA do not keep official membership records, it is not possible to report overall response rate for the survey at the participant level.

Participant and stroke support group characteristics are shown in Table 1. Stroke survivors were on average 68.6 years old. The majority of participants had been a member of their stroke group for over 12 months (76%) and attended every, or nearly every, session (79%). Most stroke support groups were classed as general support groups (68%), with aphasia groups being the second most common group type (16%).

**Table 1:** Participant and stroke support group characteristics

**Participant characteristics (N = 579)**	
Age (mean, SD)	68.6 (11.0)
Age^a^ (N, %) Under 30 30 – 39 40 – 49 50 – 59 60 – 69 70 – 79 80 – 89 90 – 99 Over 100	1 (0.2) 6 (1.0) 20 (3.5) 95 (16.4) 152 (26.3) 203 (35.1) 84 (14.5) 8 (1.4) 1 (0.2)
Gender (% female)	263 (45.6)
Time since stroke^a^ (N, %) Less than 1 year 1 – 2 years 3 – 5 years 6 – 10 years Over 10 years	42 (7.5) 91 (16.2) 171 (30.5) 130 (23.2) 127 (22.6)
Perceived health status^a^ (N, %)PoorFairGoodExcellent Poor Fair Good Excellent	39 (7.0) 240 (43.1) 239 (42.9) 29 (7.0)
Lives alone (N, %)	172 (29.7)
Questionnaire version completed (N, %) Non-aphasia Aphasia	446 (77.0) 133 (23.0)
Member of group for over 12 months (N, %)	428 (76.2)
Attend every, or nearly every session (N, %)	457 (79.3)
Group volunteer (N, %)	125 (21.6)
**Stroke support group characteristics (N = 84)**	
Group meeting frequency (N, %) Weekly Fortnightly Monthly Other	37 (44.1) 22 (26.2) 23 (27.4) 2 (2.4)
Activity (N, %) Creative arts Exercise Singing Speakability/aphasia Stroke café Support Other	7 (8.3) 1 (1.2) 2 (2.4) 13 (15.5) 3 (3.6) 57 (67.9) 1 (1.2)

^a^ Missing data means totals may not equal overall response rate

Summary outcome data are presented in Table 2. The mean loneliness score was 5.31 (scored 3 to 9), with 48% rated as often lonely (scoring 6 or above). This is higher than that reported in the general population, where 19% of people aged over 50 were often lonely [48]. Participants’ identification with their stroke support group was high (mean 3.31/4).

**Table 2:** Participant summary outcome data

Summary outcome data (*N* = 579)	
Loneliness (mean, SD)	5.31 (2.0)
Loneliness, often lonely (N, %)	262 (48.1)
Stroke group identification (mean, SD)	3.31 (0.6)
Received social support (mean, SD)	7.25 (2.4)
Given social support, agree^a^ (N, %)	391 (69.3)
Control (mean, SD)	2.57 (1.1)
Self-esteem (mean, SD)	2.47 (1.3)
Identity centrality as a stroke survivor, agree^a^ (N, %)	423 (75.5)
Goal Clarity, agree^a^ (N, %)	379 (67.4)
Group Autonomy, agree^a^ (N, %)	429 (75.9)
Same people come to every session, agree^a^ (N, %)	365 (64.5)

^a^Reference category (disagree/neither agree nor disagree)

### Relationship between stroke group identification, and loneliness (H1)

To explore the relationship between stroke support group identification and loneliness, support group identification and potential demographic covariates (age, gender, living alone, time since stroke, perceived health, volunteer status, length of group membership, group meeting frequency, and frequency of attendance) were each entered as a univariable predictor for loneliness. In the univariable models, support group identification was significantly negatively associated with loneliness (estimates for social identification and potential covariates can be found in Table 1 in Appendix B). Table 3 presents the estimated effects from the multivariable model adjusted for covariates, fitted to loneliness. In the multivariable model, the association between support group identification and loneliness remained significant after adjusting for age, living alone, perceived health, time since stroke, and volunteer status (covariates found to be significant in univariable analyses). In this model the covariates age, living alone, and perceived health also remained significant.

**Table 3:** Unstandardized B regression coefficients from a multivariable model of the relationship between support group identification and loneliness (*n* = 507)

**Predictor**	B Co-efficient	95% Confidence Interval	*P*-value
Support group identification	-0.45	-0.70 – -0.19	< 0.001***
Age (years)	-0.02	-0.04 – -0.01	0.002**
Live alone	0.35	-0.001 – 0.71	0.050*
Perceived health			
Poor/Fair	Ref	Ref	Ref
Good/Excellent	-0.75	-1.08 – -0.43	< 0.001***
Time since stroke (years)			
0 – 2	Ref	Ref	Ref
3 – 5	0.05	-0.40 – 0.50	0.826
6 – 10	-0.06	-0.56 – 0.43	0.797
< 10	-0.22	-0.72 – 0.27	0.374
Volunteer	-0.36	-0.78 – 0.06	0.092

^*^p < 0.05, **p < 0.01, ***p < 0.001

### Factors associated with stroke group identification (H2)

Table 4 presents the adjusted estimated effects for associations between psychological resources, functional processes, and strength of stroke group identification. Each group resource (given and received social support, control, self-esteem, identity centrality), function (goal clarity, group autonomy, group member continuity), and potential demographic covariates (age, gender, living alone, time since stroke, perceived health, volunteer status, length of group membership, group meeting frequency, and frequency of attendance), were first entered as univariable predictors of group identification. In these unadjusted univariable models, all group resources and functions were positively associated with stroke group identification (estimates can be found in Table 2 in Appendix B). In the multivariable model, only given and received social support, goal clarity and member continuity remained significantly positively associated, after adjusting for length of time in group, frequency of group meeting, and frequency of attendance. In summary, of all the variables predicted to be associated with group identification, it was given and received support, goal clarity and member continuity that were most closely linked with stronger identification.

**Table 4:** Unstandardized B regression coefficients from a multivariable model of the relationships between resource and function variables associated with identification (*n* = 506)

**Predictor**	B Co-efficient	95% Confidence Interval	*P*-value
Received social support	0.10	0.08 – 0.12	< 0.001***
Given social support	0.17	0.07 – 0.29	0.001**
Control	0.03	-0.02 – 0.08	0.180
Self-esteem	0.01	-0.03 – 0.06	0.554
Stroke survivor identity centrality	0.006	-0.10 – 0.11	0.914
Goal clarity	0.17	0.06 – 0.27	0.002**
Group autonomy	0.07	-0.05 – 0.19	0.241
Member continuity	0.19	0.10 – 0.29	< 0.001***
Length of time in group			
< 12 months	Ref	Ref	Ref
≥ 12 months	0.09	-0.02 – 0.19	0.116
Frequency group meeting			
Weekly	Ref	Ref	Ref
Fortnightly	-0.11	-0.22 – 0.005	0.061
Monthly	-0.09	-0.21 – 0.02	0.117
Other	0.07	-0.19 – 0.34	0.577
Frequency attendance			
Less than nearly every session	Ref	Ref	Ref
Every, or nearly every session	0.03	-0.08 – 0.14	0.604

^*^p < 0.05, **p < 0.01, ***p < 0.001

## Discussion

Whilst past research indicates that stroke survivors experience community groups as important for their ability to cope and adjust to life after stroke [13, 14], the current study extends on this to establish *how* these groups might help to shape these important outcomes. In particular, greater identification with the stroke support group was found to be associated with lower levels of loneliness. It was also found that greater given and received social support, goal clarity, and member continuity were the key group resources and functions that were associated with greater levels of identification.

These findings are in line with a central tenet of the Social Identity Approach to Heath; namely that social identification is a critical variable underpinning the health benefits of group participation. Speaking to the first research question, the association between stroke group identification and reduced loneliness, participants who identified more strongly with their stroke support group were significantly less lonely, after accounting for age, living alone, perceived health status, time since stroke, and whether they were a group volunteer. This finding is consistent with other expected health benefits of group identification, that include reduced depression and anxiety [29, 35, 49, 50]. Of note, many previous studies examining this relationship have measured identification at the community-level [51, 52], or examined the benefits of identification with multiple groups [53]. This study highlights that lower levels of loneliness are not just about multiple or wider community group belonging but can also be associated with identification with a single meaningful group, in this case a stroke support group. These benefits remained significant even after accounting for known risk factors for loneliness such as poor health and living alone [54, 55], which were also found to be associated with greater loneliness. Wakefield et al. reported similar health-enhancing effects of support group identification in people with multiple sclerosis, where identification was significantly associated with lower levels of depression and anxiety [56]. Particularly for people with chronic health conditions, this effect of support group identification on loneliness and health could be attributed to these groups offering specific resources necessary to handle these challenges, which are uniquely attainable through connections between people who share similar experiences [57].

The second research question concerned the factors that are associated with group identification in the stroke support group context. According to the social identity approach and its extension to health, the benefits of stroke support group membership (in this study, lower levels of loneliness) are predicted to be especially marked to the extent that identification is strong. Accordingly, it is important to understand the circumstances under which they are more likely to identify strongly. We hypothesised that the group resources of social support (received and given), personal control, self-esteem, and identity centrality, and group functions of group goals, autonomy, and member continuity would be associated with greater stroke support group identification. This hypothesis was partially supported, with results showing that those group members who reported high levels of received social support, given social support, goal clarity and member continuity more strongly identified with their support group. This effect was found even after controlling for how long members had been in the group, how often the group met, and how often they attended group meetings.

The importance of group-derived social support aligns with several other studies (e.g., [58, 59]. Other research has found that provision, rather than receipt, of social support is associated with greater satisfaction in retirement [60] and in stroke support groups [61, 62]. However, this “helper effect” is not found universally within peer support contexts. A randomised controlled trial of an online support group for breast cancer survivors assigned participants to a “prosocial” arm, involving tips on how to support others online, prompts for writing advice for other members, and facilitators that promoted helping behaviours. The study found that, despite offering a greater amount of social support to others, these participants experienced higher levels of anxiety and depression than those assigned to a standard online support group, with no differences between trial arms in the perception of helpfulness of the group [63]. Although data are mixed in the wider support group literature, in the present stroke support group context given social support was associated with stronger group identification. Further research exploring social identification as a potential mechanism may help to better understand the conditions underlying this helper effect in support groups more broadly and help avoid unintended consequences of prosocial manipulation.

However, the other psychological resources measured here – personal control, self-esteem, and stroke survivor identity centrality – were not found to be associated with group identification in controlled analysis. As these processes were significant as single predictors, it may be that a degree of intercorrelation was present and that, as the strongest predictor, social support masked the effects of the other variables. This may be because social support was explicitly stated as the main purpose of attending the groups studied here; thus, where people identify with the group they are subscribing to and engage with support as the central tenet of the group. Whereas personal control and self-esteem have previously been identified as important predictors for depression [64] and quality of life [65] in stroke survivors, these were not necessarily resources that were advertised as explicit benefits of attending, and were not associated here with identification with the stroke group.

The finding that identity centrality as a stroke survivor was not related to group identification was inconsistent with research findings in other health conditions. For example, among participants in obesity and body image treatment groups, for example, greater integration of weight [36] and the thin ideal [66], respectively, into one’s self-concept contributed to stronger identification with these treatment groups. Similar effects have been reported with self-identified cancer survivors who participate in online support groups [67]. Our findings were also inconsistent with qualitative research, where stroke survivors frequently acknowledge that adjusting to a new sense of self is important for post-stroke wellbeing [68, 69]. Woodman, Riazi [70] suggest that acceptance of stroke-related limitations is a key factor in social participation after stroke, and that support from other stroke survivors helped move individuals onto a positive adjustment trajectory [71]. It may be that whilst identity as a stroke survivor is an important gateway to accessing support from a group, in the established groups such as those sampled here, that sense of self as a stroke survivor is not as relevant to ongoing identification as is social support. Furthermore, whilst identity as a stroke survivor may be important in daily life, it seems possible that the identity that is salient during support group meetings may instead be that which is derived directly from the group. This salience may have been emphasised during questionnaire data collection where participants were primed to think about their stroke group membership.

Turning to group function, goal clarity and group membership continuity were found to be associated with identification. The importance of having the same people attend each session to build a strong sense of connection is consistent with other research [36], and is intuitive in the sense that meeting the same people each week gives rise to greater familiarly between members and ease of connection. Goals have previously been explored as a dimension of group relationships in psychotherapy settings, where member–member and member-leader agreement on therapeutic tasks and goals emerged as a distinct factor contributing to how members experience group relationship quality [72]. The present research supports the idea that goals are also important for shared identification. Group autonomy was not associated with support group identification. Group-relevant decision making has been used as a method of building social identification in numerous interventions that have been significantly associated with better health [35]. Although three-quarters of the participants in the current study agreed that they could influence the content and outcomes of the group sessions, it was unclear whether this was seen as a benefit of stroke group participation which would be needed to strengthen their connection to the group. Qualitative research with support group members could help understand this finding, to provide greater focus when exploring this concept in future research.

### Practical recommendations and future research

The findings from this study have implications for the delivery of support groups aiming to promote and embed a sense of social identity. The resources and functions found here to be significantly associated with support group identification may be influenced and shaped not just by the actions of those within the group but also by the group’s design. For example, social support (as the primary purpose of the group) can be provided through direct group facilitation, or it may be encouraged in others, and facilitators can also help to clarify and collectively refine the group’s goals [73]. Furthermore, continuity of group membership is a dynamic variable that can nonetheless be shaped through the organisation of the group – it promotes a “closed” group environment which can result in greater cohesion than open groups [74]. Although in the present study the group membership was mostly stable, as most respondents attended every or nearly every session, support groups often need to operate in an “open” format in order to be able to offer their services to the many stroke survivors and to allow for natural turnover. Practically, this may mean facilitators need to be alert to the impact of new members joining the group and take steps to enable early integration of these with existing members; for example, through dedicated group activities. Indeed, research has indicated that some activities, such as group singing, can encourage rapid identity formation amongst members faster than those who take part in individual activities such as arts-based craft or creating writing groups [75]. Whilst the present study collected data on group activity type, there was insufficient variance to test whether this variable was associated with social identification.

The study also provides direction for future research in this field. Building on these cross-sectional findings from established groups, future research should focus on using longitudinal methods to examine how these processes develop from new groups, and how the relative importance of different processes change as the group matures. Furthermore, a clear aim for future applied research is to understand how these processes can be *actively* managed to bring about positive health outcomes in applied intervention settings. The answer may lie in intervention design and facilitation that creates an environment where these processes can flourish. The Social Identity Model of Behaviour Change [73] may help guide this process, as it was developed for the purpose of raising recognition of these group resources and functions (including those addressed in RQ2) as a key mechanism supporting behaviour change in group-based interventions. Finally, as most participants in this study were established group members, future research should investigate the experiences of those who started attending these groups but subsequently dropped out, with particular attention to how group processes might have shaped their engagement with the group.

### Strengths and limitations

This study was conducted using a large, national dataset of people with stroke. A general limitation is that some measures used non-validated single items; this approach was chosen in order to measure a large number of processes whilst minimising burden to stroke survivors. Furthermore, an adapted questionnaire was available which meant this study was inclusive of people with aphasia, who are often excluded from research. Adapting the survey for people with aphasia meant that a subset (23%) of the data did not use a validated scale. Concerns about common method bias may be valid here because of the similarities in the way the measures were administered. However, techniques to avoid common method bias such as varying the anchors of a Likert scale would be problematic for this population, adding additional cognitive burden. As such, these limitations can be considered an acceptable trade-off for improvements in generalisability of the stroke population, of which 40% have aphasia [76]. However, given the limitations of non-validated measures (i.e., that they may not accurately measure the construct they aim to capture), these results should be interpreted with caution.

The initial plan for this project was to conduct a longitudinal analysis of stroke support groups, but this was not possible due to the COVID-19 pandemic. We therefore cannot comment on the temporal relationship between social identity, group processes, and wellbeing outcomes. Whilst our aim here was not to examine directionality, the fluidity of group identification, which may evolve and change over time, should be recognised [24, 77, 78]. Nonetheless, by examining group resources and functions arising from group identification in established groups, taking a cross-sectional overview may aid identification of important identity-relevant processes to be prioritised for further exploration in longitudinal studies.

The self-selecting nature of the participants studied here may mean only successful groups responded to the survey. This is further evident in the skewed nature of some of the process measures which required dichotomisation. Finally, the range of cluster (group) sizes meant we did not model within or between group differences of the relationship of group processes on outcomes.

### Conclusions

Stroke support group identification may play an important role in reducing loneliness for stroke survivors that attend stroke support groups. A range of group resources (given and received social support) and functions (goal clarity, and group member continuity) were particularly strongly associated with greater support group identification. These findings contribute to the body of knowledge illustrating how support groups exert their effects. Theoretical alignment with the Social Identity Approach to Heath means these findings can be compared to and inform interventions that might be designed following that approach. Future research using longitudinal methods may help further disentangle the directionality and temporal effects of these processes and outcomes.

## Supplementary Information

Supplementary Material 1

Supplementary Material 2

## Data Availability

The datasets analysed during the current study are not publicly available, as participants consented to sharing their anonymised data solely with approved researchers through an independent ethics committee. Data are available from the corresponding author upon reasonable request and with appropriate ethical approval. For the purpose of open access, the author has applied a Creative Commons Attribution (CC BY) licence to any Author Accepted Manuscript version arising from this submission.
