# Supplementary material for: A UK national cross-sectional survey of stroke support groups: exploring the role of social identification and group processes in reducing loneliness
Source: BMC Public Health. 2024 Oct 29;24:2992. doi: 10.1186/s12889-024-20432-w (PMC11520689; doi:10.1186/s12889-024-20432-w)
Supplement: Supplementary file 1 — Supplementary Material 1 [file 12889_2024_20432_MOESM1_ESM.docx]

**Appendix A**: Exploratory multiple imputation of missing variables

Data were assumed missing at random. No auxiliary variables were added to the model. Models were run using the ‘mi impute’ function in Stata 18 using the multivariate normal regression method.

| **Table A1**  Unstandardized B coefficients of the relationship between support group identification, resources arising from social identification, and loneliness | | | | | | | |
| --- | --- | --- | --- | --- | --- | --- | --- |
|  | **Univariable** | | | | **Multivariable Model** | | |
| Predictor | B Co-efficient | 95% CI | P-value | B Co-efficient | | 95% CI | P-value |
| Support group identification | -0.52 | -0.77 – -0.26 | **<0.001***** | -0.45 | | -0.69– -0.20 | **<0.001***** |
| Age (years) | -0.02 | -0.04 – -0.01 | **0.003**** | -0.02 | | -0.04 – -0.01 | **0.001**** |
| Gender (female) | 0.14 | -0.19 – 0.47 | 0.406 |  | |  |  |
| Live alone | 0.46 | 0.11 – 0.82 | **0.011*** | 0.37 | | 0.03 – 0.72 | **0.035*** |
| Perceived health  Poor/Fair  Good/Excellent | Ref  -0.79 | Ref  -1.09 – -0.47 | Ref  **<0.001***** | Ref  -0.68 | | Ref  -0.98 – -0.37 | Ref  **<0.001***** |
| Time since stroke (years) | -0.17 | -0.32 – -0.01 | **0.035*** | -0.09 | | -0.25 – 0.06 | 0.236 |
| Volunteer | -0.41 | -0.81 – -0.01 | **0.045*** | -0.29 | | -0.70 – 0.12 | 0.171 |
| Length of time in group  < 12 months  ≥ 12 months | Ref  0.03 | Ref  -0.25 – 0.30 | Ref  0.856 |  | |  |  |
| Frequency group meeting | -0.06 | -0.28 – 0.15 | 0.575 |  | |  |  |
| Frequency attendance  Less than nearly every session  Every, or nearly every session | Ref  -0.07 | Ref  -0.45 – 0.31 | Ref  0.716 |  | |  |  |

| **Table A2**  Variables associated with identification | | | | | | | | | |  |  |  |
| --- | --- | --- | --- | --- | --- | --- | --- | --- | --- | --- | --- | --- |
|  | | **Univariable** | | | | **Multivariable** | | |  |  |  |  |
| Predictor | B Co-efficient | | 95% CI | P-value | B Co-efficient | | 95% CI | P-value |  |  |  |  |
| Received social support | 0.15 | | 0.13 – 0.17 | **<0.001***** | 0.11 | | 0.09 – 0.13 | **<0.001***** |  |  |  |  |
| Given social support | 0.33 | | 0.22 – 0.43 | **<0.001***** | 0.11 | | 0.01 – 0.20 | **0.025*** |  |  |  |  |
| Control | 0.11 | | 0.07 – 0.16 | **<0.001***** | 0.03 | | -0.02 – 0.07 | 0.227 |  |  |  |  |
| Self-esteem | 0.10 | | 0.05 – 0.14 | **<0.001***** | 0.02 | | -0.02 – 0.06 | 0.289 |  |  |  |  |
| Stroke survivor identity centrality | 0.06 | | -0.04 – 0.16 | 0.221 |  | |  |  |  |  |  |  |
| Goal clarity | 0.34 | | 0.24 – 0.44 | **<0.001***** | 0.13 | | 0.03 – 0.22 | **0.008**** |  |  |  |  |
| Group autonomy | 0.29 | | 0.18 – 0.40 | **<0.001***** | 0.03 | | -0.07 – 0.12 | 0.604 |  |  |  |  |
| Member continuity | 0.37 | | 0.26 – 0.47 | **<0.001***** | 0.22 | | 0.13 – 0.31 | **<0.001***** |  |  |  |  |
| Age (years) | 0.002 | | -0.002 – 0.01 | 0.324 |  | |  |  |  |  |  |  |
| Gender (female) | 0.08 | | -0.03 – 0.18 | 0.139 |  | |  |  |  |  |  |  |
| Live alone | -0.05 | | -0.17 – 0.06 | 0.374 |  | |  |  |  |  |  |  |
| Perceived health  Poor/Fair  Good/Excellent | Ref  0.07 | | Ref  -0.04 – 0.17 | Ref  0.197 |  | |  |  |  |  |  |  |
| Time since stroke (years) | 0.04 | | -0.01 – 0.09 | 0.133 |  | |  |  |  |  |  |  |
| Volunteer | 0.03 | | -0.10 – 0.16 | 0.678 |  | |  |  |  |  |  |  |
| Length of time in group  < 12 months  ≥ 12 months | Ref  0.11 | | Ref  0.03 – 0.20 | Ref  **0.008**** | 0.08 | | 0.01 – 0.14 | **0.031*** |  |  |  |  |
| Frequency group meeting | 0.04 | | -0.01 – 0.09 | 0.133 | -0.04 | | -0.09 – 0.01 | 0.114 |  |  |  |  |
| Frequency attendance  Less than nearly every session  Every, or nearly every session | Ref  0.20 | | Ref  0.08 – 0.33 | Ref  **0.001**** | Ref  0.03 | | Ref  -0.07 – 0.14 | Ref  0.510 |  |  |  |  |
