# Supplementary material for: A UK national cross-sectional survey of stroke support groups: exploring the role of social identification and group processes in reducing loneliness
Source: BMC Public Health. 2024 Oct 29;24:2992. doi: 10.1186/s12889-024-20432-w (PMC11520689; doi:10.1186/s12889-024-20432-w)
Supplement: Supplementary file 2 — Supplementary Material 2 [file 12889_2024_20432_MOESM2_ESM.docx]

| **Table B1**  Unstandardized B regression coefficients from univariable models of the relationship between support group identification and loneliness | | | | | | |
| --- | --- | --- | --- | --- | --- | --- |
| **Predictor** | B Co-efficient | 95% Confidence Interval | | P-value | |  |
| Support group identification | -0.52 | -0.77 – -0.26 | | **<0.001***** | |  |
| Age (years) | -0.02 | -0.04 – -0.01 | | **0.003**** | |  |
| Gender (female) | 0.14 | -0.19 – 0.47 | | 0.405 | |  |
| Live alone | 0.46 | 0.11 – 0.82 | | **0.011*** | |  |
| Perceived health  Poor/Fair  Good/Excellent | Ref  -0.90 | Ref  -1.23 – -0.58 | | Ref  **<0.001***** | |  |
| Time since stroke (years)  0 – 2  3 – 5  6 – 10  < 10 | Ref  -0.12  -0.26  -0.47 | Ref  -0.57 – 0.34  -0.76 – 0.23  -0.95 – 0.02 | | Ref  0.617  0.289  0.058 | |  |
| Volunteer | -0.41 | -0.81 – -0.01 | | **0.045*** | |  |
| Length of time in group  < 12 months  ≥ 12 months | Ref  -0.25 | Ref  -0.64 – 0.14 | | Ref  0.201 | |  |
| Frequency group meeting  Weekly  Fortnightly  Monthly  Other | Ref  -0.01  -0.21  0.10 | Ref  -0.48 – 0.47  -0.69 – 0.28  -1.04 – 1.24 | | Ref  0.979  0.409  0.861 | |  |
| Frequency attendance  Less than nearly every session  Every, or nearly every session | Ref  -0.08 | Ref  -0.49 – 0.32 | | Ref  0.689 | |  |
| * = p < 0.05, ** = p < 0.01, *** = p < 0.001 | | |  | |  | |

**Appendix B:** univariable analysis between identification and loneliness, and between resources and function variables and identification

Table B1 presents the results of univariable (unadjusted) analyses between support group identification and potential demographic covariates (age, gender, living alone, time since stroke, perceived health, volunteer status, length of group membership, group meeting frequency, and frequency of attendance) and loneliness. Support group identification, age, having good or excellent perceived health, and being a volunteer were significantly negatively associated with loneliness. Living alone was significant positively associated with loneliness.

| **Table B2**  Unstandardized B regression coefficients from univariable models of the relationships between resource and function variables associated with stroke group identification | | | |
| --- | --- | --- | --- |
| **Predictor** | B Co-efficient | 95% Confidence Interval | P-value |
| Received social support | 0.15 | 0.13 – 0.17 | **<0.001***** |
| Given social support | 0.46 | 0.36 – 0.57 | **<0.001***** |
| Control | 0.11 | 0.07 – 0.16 | **<0.001***** |
| Self-esteem | 0.10 | 0.05 – 0.14 | **<0.001***** |
| Stroke survivor identity centrality | 0.16 | 0.04 – 0.28 | **0.009**** |
| Goal clarity | 0.49 | 0.38 – 0.59 | **<0.001***** |
| Group autonomy | 0.39 | 0.27 – 0.51 | **<0.001***** |
| Member continuity | 0.37 | 0.27 – 0.48 | **<0.001***** |
| Age (years) | 0.002 | -0.002 – 0.01 | 0.323 |
| Gender (female) | 0.08 | -0.03 – 0.18 | 0.139 |
| Live alone | -0.05 | -0.17 – 0.06 | 0.374 |
| Perceived health  Poor/Fair  Good/Excellent | Ref  0.07 | Ref  -0.03 – 0.18 | Ref  0.177 |
| Time since stroke (years)  0 – 2  3 – 5  6 – 10  < 10 | Ref  0.10  0.12  0.12 | Ref  -0.05 – 0.24  -0.04 – 0.27  -0.04 – 0.28 | Ref  0.189  0.138  0.132 |
| Volunteer | 0.03 | -0.10 – 0.16 | 0.678 |
| Length of time in group  < 12 months  ≥ 12 months | Ref  0.23 | Ref  0.10 – 0.36 | Ref  **<0.001***** |
| Frequency group meeting  Weekly  Fortnightly  Monthly  Other | Ref  -0.27  -0.17  0.09 | Ref  -0.43 – -0.12  -0.33 – -0.01  -0.29 – 0.48 | Ref  **0.001****  **0.038***  0.635 |
| Frequency attendance  Less than nearly every session  Every, or nearly every session | Ref  0.21 | Ref  0.08 – 0.34 | Ref  **0.001**** |
| * = p < 0.05, ** = p < 0.01, *** = p < 0.001 | |  |  |

Table B2 presents the results of univariable (unadjusted) analyses of the relationship between resources and function variables, and potential demographic covariates, with stroke group identification. All resources (received and given social support, control, self-esteem, and stroke survivor identity) and function variables (goal clarity, group autonomy, and member continuity) were significantly positively associated with stroke group identification. Demographic variables length of time in group, frequency of group meeting and frequency of attendance were also significantly associated with stroke group identification.
